# The Effect of Leisure Intervention on Occupational Performance and Occupational Balance in Individuals with Substance Use Disorder: A Pilot Study

**DOI:** 10.1155/2024/6299073

**Published:** 2024-02-14

**Authors:** Majid Farhadian, Malahat Akbarfahimi, Peyman Hassani Abharian, Mitra Khalafbeigi, Farzaneh Yazdani

**Affiliations:** ^1^School of Rehabilitation Sciences, Iran University of Medical Sciences, Tehran, Iran; ^2^Department of Occupational Therapy, School of Rehabilitation Sciences, Neuroscience Research Center, Iran University of Medical Sciences, Tehran, Iran; ^3^Institute for Cognitive Science Studies (ICSS), Tehran, Iran; ^4^Occupational Therapy Program, Faculty of Health and Life Sciences, Oxford Brookes University, Oxford, UK

## Abstract

**Methods:**

The sample for this quasiexperimental pretest–posttest with a two-month follow-up design comprised nine individuals aged between 18 and 55 years, selected using a convenience sampling method. The intervention consisted of a 2-month group leisure participation program, conducted twice a week, followed by a 2-month follow-up period. Primary outcome measures included occupational performance and occupational balance, and secondary outcome measures were leisure participation, quality of life, and drug craving. Outcome measures were assessed three times: preintervention, postintervention, and after the follow-up period. The outcome measures included the Canadian Occupational Performance Measure (COPM), Occupational Balance Questionnaire-11 (OBQ11), Nottingham Leisure Questionnaire (NLQ), 36-Item Short-Form Health Survey (SF-36), and Desire to Drug Questionnaire (DDQ). Data analysis was performed using the Friedman test and Wilcoxon signed-rank test as a post hoc procedure, with a significance level set at 5%.

**Results:**

The findings showed significant improvements in participants' occupational performance in postintervention and follow-up assessments (*p* < 0.01, *r* = 0.59) and better occupational balance from pre- to postintervention (*p* < 0.01, *r* = 0.59) and after the follow-up period (*p* < 0.01, *r* = 0.60). Furthermore, significant enhancements were observed in leisure participation, quality of life, and a reduction in drug craving.

**Conclusion:**

The findings indicate that leisure intervention positively impacted both occupational performance and occupational balance, suggesting its potential as a beneficial therapeutic approach for individuals with substance use disorder. Additional research is warranted to delve deeper into and validate the effectiveness of leisure intervention within this specific population.

## 1. Introduction

Substance use disorder is currently recognized as one of the most significant global issues. It gives rise to a range of physical, cognitive, and behavioral impairments [1, 2]. Despite the awareness of these substantial problems associated with substance use, individuals with substance use disorder persist in their substance consumption [3], leading to limitations in activities and social engagement that negatively impact their occupational and overall quality of life [4]. For individuals with substance use disorder, substance consumption becomes a central component of their daily routines and contributes to the development of a substance-dependent identity [5]. Consequently, individuals in recovery from substance use face the loss of occupations that once constituted a core part of their daily lives and identity. This loss may subsequently lead to reduced treatment adherence and an increased risk of relapse [5]. Occupation limitations are characteristic of individuals with substance abuse disorder, as substance use significantly affects time management, engagement in meaningful daily occupations, and fulfillment of essential life roles, ultimately resulting in occupational imbalances [6]. The lack of diverse occupational opportunities further contributes to increased substance use, while ineffective time utilization and poor habits affect occupational performance and balance across different environments [5]. Research has demonstrated that individuals with substance use disorder experience occupational challenges and imbalances that should be considered during intervention processes.

There are evidence-based interventions for substance use disorder treatment, including brief interventions, cognitive behavioral therapy, motivational strategies, and 12-step programs [7]. Given the evidence that individuals with substance use disorder face reduced occupational participation, therapeutic interventions that emphasize the use of occupation as a means of service delivery are necessary. Among various occupations, leisure can be regarded as a key area for interventions during periods of abstinence, as leisure time is often diminished and undergoes changes in meaning and execution among most individuals with substance use disorder [8]. Leisure occupations significantly impact individuals' lives and are defined as nonmandatory activities pursued with internal motivation during free time, distinct from work, self-care, or sleep [9]. Participating in leisure activities fulfills crucial psychological needs such as a sense of belonging, self-esteem, stimulation, self-expression, creativity, and competition. Leisure is closely tied to quality of life, promoting life balance, health, and overall life satisfaction [10]. Many individuals struggling with substance use disorder experience boredom during leisure time before engaging in substance use and face challenges in selecting appropriate leisure activities and deriving enjoyment from them. In many cases, substances are used to initiate, facilitate, or enhance participation or pleasure in leisure activities [11] or even as a leisure pursuit itself [12]. Following periods of abstinence, individuals often have a surplus of free time, which increases the likelihood of engaging in high-risk activities and subsequent substance use [13].

Moreover, it is important to note that participation in leisure occupations activates the brain's reward system [14]. This is particularly significant as substance use disorder is associated with pronounced changes in the brain's reward system, leading to frequent cravings and a strong propensity for substance recurrence when individuals are exposed to substance-related stimuli [3]. Enjoyable activities and leisure pursuits stimulate the brain's reward system and trigger the release of dopamine, similar to how addictive substances induce stimulation and elevate dopamine levels in the mesocorticolimbic system [15]. Engaging in occupations characterized by enjoyment, social interaction, and cognitive and physical stimulation can serve as an optimal means of creating meaning, fostering a sense of well-being, and promoting recovery in life [16].

Given the aforementioned challenges in occupational performance among individuals with substance use disorders, the positive effects of leisure participation, and the strong association between leisure and substance use disorder with the brain's reward system, it can be argued that leisure occupations constitute an area of intervention for occupational therapists in the realm of substance use disorder. Hence, the aim of this study was to investigate the feasibility of leisure interventions for individuals with substance use disorder and to provide preliminary data for use in future randomized controlled trials.

## 2. Methods

### 2.1. Study Design

This pilot study employed a quasiexperimental pretest–posttest design with a 2-month follow-up and was conducted between September 2022 and March 2023. The research protocol obtained approval from the Ethics Committee of Iran University of Medical Sciences (IR.IUMS.REC.1401.623). Comprehensive information regarding the study's objectives, potential benefits, and associated risks was provided to the participants. Assurance of voluntary participation and the freedom to withdraw from the study at any point was conveyed. Subsequently, participants were administered the study instruments and intervention after obtaining signed consent forms, which were approved by the Ethics Committee of Iran University of Medical Sciences.

### 2.2. Setting and Participants

A target sample of 12 subjects was initially chosen for this pilot study based on evidence suggesting that a sample size of 12 participants is ideal for preliminary studies [17]. However, during the research process, three participants were excluded. Two individuals were absent for more than four intervention sessions, and one person opted not to participate in the postintervention evaluations. Consequently, a convenience sample of nine individuals with substance use disorder (SUD) ultimately participated in the study. To identify potential participants for the study, the welfare organization of Tehran was initially contacted. A list of drug treatment outpatient clinics, local support groups, and Narcotics Anonymous communities was obtained from this organization. Subsequently, the researcher visited these centers, explained the research process, and selected individuals who expressed interest and volunteered to participate in the study.

All assessments, as well as sessions 1, 2, and 12 of the interventions, took place at the Chavan Occupational Therapy Center. The remaining sessions were conducted at the Azadi Complex in Tehran.

Upon the study's conclusion, participants received a comprehensive report summarizing their performance in the assessments, providing them with feedback on their involvement in the research.

Inclusion criteria required participants to meet the Diagnostic and Statistical Manual of Mental Disorders, 5th Edition (DSM-5) criteria for substance use disorders, not undergoing concurrent pharmacological treatments, does not have comorbid psychiatric disorders according to DSM-5 criteria, have no history of neurological conditions or brain scan abnormalities, and have at least 15 days of current abstinence from all substances of abuse, including alcohol, to rule out withdrawal symptoms. Regular weekly urinary tests were conducted to ensure participants maintained abstinence throughout the study period.

### 2.3. Intervention

The intervention consisted of 12 sessions conducted twice a week in a group setting, facilitated by an occupational therapist. The objective of the intervention was to familiarize participants with the concept of leisure, along with identifying both barriers and enablers related to it. The aim was to foster an understanding of preferred and accessible leisure activities, ultimately facilitating the process of leisure planning and engagement. The initial two and final sessions focused on education and planning, familiarizing participants with leisure concepts, identifying barriers and facilitators to leisure, understanding preferred and available leisure activities, and developing personalized leisure plans. The remaining sessions [3–11] involved practical activities where the group engaged in chosen leisure occupations together. A comprehensive list of leisure occupations was initially compiled based on a literature review, considering evidence and documentation in the field of leisure occupations. Participants' interests, as determined by the Nottingham Leisure Questionnaire, were also taken into account. Expert opinions and qualitative evaluations, including interviews and the Canadian Occupational Performance Measure (COPM), further informed the selection of leisure occupations. The group was given the opportunity to suggest additional favorite leisure activities not on the list. The chosen leisure activity with the highest group preference was considered the therapeutic intervention and implemented according to the predetermined schedule.  Table 1 provides a detailed outline of the steps involved in implementing the leisure interventions.

### 2.4. Outcome Measures

The study comprised three assessment points: a baseline preintervention assessment, a postintervention assessment conducted after 8 weeks of completing the leisure time intervention, and a final assessment following an 8-week follow-up period.

### 2.5. Primary Outcome Measures

#### 2.5.1. Canadian Occupational Performance Measure (COPM)

This semistructured interview-based tool enables occupational therapists to assess changes in clients' perceived occupational performance and satisfaction over time. It comprises three domains: personal care, productivity, and leisure. Participants were instructed to employ a 10-point scale to assess their present performance and satisfaction regarding each identified issue. The assessor determined the COPM performance and satisfaction score by dividing the total assigned score by the number of identified problems. In the subsequent assessment, individuals once again rated their performance and satisfaction levels for the same issues. Therapists utilized these scores to compute a change score, with an increase indicating improved performance and satisfaction. Trained therapists conducted the COPM interviews, which typically lasted 20-30 minutes [17].

#### 2.5.2. Occupational Balance Questionnaire (OBQ11)

This 11-item questionnaire measures occupational balance, defined as the appropriate distribution and diversity of occupations. Each item is scored on a 0-3 scale, with higher scores indicating better occupational balance. The OBQ11 has demonstrated high reliability (Cronbach's alpha = 0.92) [18].

### 2.6. Secondary Outcome Measures

#### 2.6.1. Nottingham Leisure Questionnaire (NLQ)

This self-report questionnaire consists of 30 Likert-scale items (range: 0-2) corresponding to different leisure activities. An additional “other” category allows participants to add activities not listed. The NLQ's test-retest reliability has been acceptable (kappa = 0.44 to 0.94), and the French version demonstrated satisfactory internal consistency (*α* = 0.76) [19, 20]. The researchers added two items (playing video/multimedia games and Internet-based activities) to reflect contemporary leisure preferences.

#### 2.6.2. 36-Item Short-Form Health Survey (SF-36)

This survey assesses health-related quality of life and comprises physical and mental health subscales with a total of 8 subscales. Scores range from 0 to 100, with higher scores indicating better quality of life. The SF-36 has reported reliability coefficients of 0.77 to 0.95 [21].

#### 2.6.3. Desire to Drug Questionnaire (DDQ)

Derived from the alcohol craving scale and modified for measuring cravings across various substances, the 14-item DDQ assesses current drug cravings using a 7-point Likert scale, and higher scores indicate higher craving for drugs. It examines desire to use drugs, negative reinforcement, and perceived control over drug use. The DDQ has shown good reliability, with Cronbach's alpha coefficients of 0.89, 0.79, and 0.4 for each factor, respectively [22].

### 2.7. Statistical Analysis

Descriptive statistics, including means, frequencies, and standard deviations, were employed to summarize the data. Given the small sample size and ordinal level of the data, Friedman's nonparametric analysis was utilized to assess differences between the preintervention, postintervention, and follow-up assessments for each outcome measure. The Wilcoxon signed-rank test (two-tailed) was applied as a post hoc analysis to evaluate changes after each assessment period. Given the utilization of multiple instruments, the *p* value adjustment method was applied to control for type I error. This entailed dividing the original *p* value (0.05) by the number of assessments conducted, resulting in an adjusted *p* value of 0.016 for both primary and secondary outcome measures. Effect sizes (*r*) were calculated for the post hoc Wilcoxon signed-rank tests by dividing the *z*-score by the square root of the total number of observations (*N*) [23]. Effect sizes were interpreted according to Cohen's criteria as small (*r* ≥ 0.10), medium (*r* ≥ 0.30), or large (*r* ≥ 0.50) [24]. Statistical analyses were performed using IBM SPSS Statistics (version 23).

## 3. Results

A total of nine participants completed the intervention and all phases of assessment. The participants had a mean age of 30.11 years (SD = 7.99), with a mean duration of drug use of 4.66 years (SD = 2) and an average abstinence period of 21 months (SD = 10.75). The demographic characteristics of the participants are summarized in Table 2.

No unanticipated adverse events were reported during the intervention phase. The mean scores of the outcome measures at each assessment are presented in Table 3.

According to the results of the analysis, significant differences were identified across the three conditions (preintervention, postintervention, and follow-up) for all variables (*p* value < 0.001).

Subsequently, the Wilcoxon signed-rank test analysis was performed to further examine the differences between the preintervention and postintervention assessments, as well as the preintervention and follow-up assessments. As presented in Table 4, the results showed significant differences with large effect sizes (ranging from 0.59 to 0.60) between the preintervention and postintervention/follow-up assessments, indicating the effectiveness of the intervention. However, no significant differences were observed between the postintervention and follow-up assessments (*p* > 0.016).

## 4. Discussion

The current study was aimed at investigating the efficacy of a two-month leisure intervention in enhancing occupational performance, occupational balance, leisure participation, quality of life, and drug craving among individuals with substance use disorder (SUD). The study findings reveal significant differences between preintervention and postintervention assessments, as well as between preintervention and follow-up assessments. However, no significant differences were observed between postintervention and follow-up assessments. In alternative terms, active engagement in leisure activities may significantly improve occupational performance and occupational balance among this population. Additionally, the leisure intervention demonstrated positive effects on leisure participation, quality of life, and drug craving. However, it is important to note that the effect sizes decreased from postintervention to follow-up, indicating a slight diminishing of the intervention's effects over time.

The results of this pilot study showed that participating in leisure activities subsequent to drug abstinence had a profound and enduring impact on occupational performance and occupational balance, even during the two-month follow-up period when participants engaged in leisure activities individually. It has been demonstrated that individuals with substance use disorders encounter difficulties in engaging in their daily occupations while under the influence of drugs, as well as during the period of abstinence from substance use [6]. One potential avenue for instigating a positive change in the occupational life of individuals with mental health conditions may be through the utilization of leisure occupations. Prior research suggests that involving individuals in leisure occupations can serve as a means to transfer feelings of well-being and pleasure to various facets of their lives, ultimately contributing to further advancements in their recovery process [25]. Furthermore, research highlights the multifaceted benefits of leisure in fostering a meaningful life, encompassing aspects of identity, belonging, engagement, and alleviation of boredom [25]. Leisure activities also serve as effective stress management strategies and can provide individuals in recovery with novel perspectives and directions in life [26, 27]. By actively engaging in leisure activities, individuals with SUDs can experience enhanced social benefits, including expanded social networks, increased opportunities for social interaction and participation, and improved social skills [28]. The pursuit of meaningful leisure activities plays a crucial role in achieving a sense of balance in the lives of individuals with SUD [29]. Given these advantages associated with leisure occupations, it is reasonable to hypothesize that engaging in leisure activities not only reintroduces a neglected occupation into participants' daily routines but also enhances the fundamental prerequisites and skills necessary for performing other areas of occupation, ultimately resulting in improved occupational performance and occupational balance.

Although leisure was not the primary outcome measure in this study, it served as both a means and an end of the intervention. The findings demonstrated significant improvements in leisure participation among individuals with SUDs following the intervention. The nature and timing of the intervention suggested an anticipated increase in participants' engagement in leisure occupations during the postintervention assessment. Therefore, a more comprehensive evaluation of the acceptance of leisure as a meaningful and repeatable occupation among participants was possible after the follow-up period. The results of the leisure assessment conducted after the follow-up period indicated that participants continued to actively engage in leisure activities. The intervention incorporated leisure education and planning sessions, in addition to practical participation in leisure occupations. Previous studies have demonstrated that leisure education contributes to increased satisfaction and participation in leisure activities [30, 31]. Thus, the educational sessions likely familiarized participants with various leisure activities, generated interest in leisure occupations, and enhanced their knowledge about available leisure resources. Furthermore, the practical engagement in leisure occupations not only improved participants' physical and mental well-being but also elicited a sense of pleasure and motivation for sustained involvement in planned leisure activities.

Another significant finding of this research pertains to the positive impact of participating in leisure activities on individuals' quality of life following drug abstinence. Substantial evidence underscores the significance of leisure in enhancing overall quality of life. Engaging in satisfying leisure activities fulfills psychological needs and contributes to subjective well-being [32]. The availability of a diverse range of leisure occupations has been associated with a reduced risk of depression and improved mental well-being [33, 34]. Active participation in leisure activities enables individuals to cultivate social relationships, experience positive emotions, acquire new skills and knowledge, and ultimately enhance their overall quality of life [34].

Furthermore, the study revealed a decrease in drug craving among participants following their engagement in leisure activities. Drug craving is characterized as a subjective state encompassing a strong desire and motivation to use drugs [35]. The availability of meaningful occupation and the absence of idle time have been shown to reduce the likelihood of substance use relapse [36]. Active participation in leisure activities not only fosters motivation and improves occupational performance but also reduces idle time, thereby diminishing the desire to engage in drug use. These findings align with previous research, which has demonstrated that individuals with substance use disorders often perceive free time as a risk factor for relapse, whereas engaging in leisure occupations serves as an active strategy for relapse prevention [37].

## 5. Conclusion

The study findings indicate that leisure interventions could be a viable option for enhancing positive therapy outcomes, including occupational performance, occupational balance, leisure participation, quality of life, and reducing drug craving among individuals with substance use disorder. However, these findings warrant confirmation through additional studies. Future research endeavors should focus on incorporating larger sample sizes and control groups and conducting more in-depth analyses of the specific content of leisure interventions tailored for this population.

### 5.1. Limitations

Several limitations should be considered when interpreting the results of this pilot study. First, the study utilized a small sample size comprised solely of men, which limits the generalizability of the findings to broader populations of individuals with substance use disorder. Future studies should strive to include larger and more diverse samples to enhance the external validity of the findings.

Furthermore, during the course of the interventions, certain issues such as overcrowding, equipment malfunctions, and time constraints were encountered, which could have influenced the participants' experiences. To mitigate these challenges, it is recommended to have alternative leisure options readily available to ensure uninterrupted engagement in leisure activities.

It is important to note that this study employed a pre-post design without a control group, which limits the ability to establish causal relationships or determine the specific contribution of the leisure intervention to the observed improvements. Future research should employ randomized clinical trials with larger sample sizes and control groups to better understand the effectiveness of leisure interventions in individuals with substance use disorder and to assess their comparative efficacy against other interventions.

## Figures and Tables

**Table 1:** Intervention and session content.

Session 1: introduction and basic explanations about leisure
(i) Introduction of the participants and explanation of the research purpose (ii) Discussion and exchange of opinions regarding participants' views on leisure time, including its definition, benefits, disadvantages, and obstacles (iii) Summary of opinions by the occupational therapist (iv) Presentation by the occupational therapist on the importance and role of leisure activities in life, characteristics of a leisure activity, and how to choose and engage in leisure activities while considering obstacles and facilitators (v) Practical review of the discussed topics, such as writing down leisure activities and checking and exchanging opinions

Session 2: choosing and planning of leisure occupations
(i) Providing and reviewing a list of leisure time occupations (ii) Assisting participants in choosing their favorite leisure activities (iii) Facilitating an exchange of opinions among participants regarding their selected leisure activities (iv) Planning and scheduling the chosen activities (v) Recording the activities in the leisure program list

Sessions 3-11: implementation of leisure
Participants engage in their chosen leisure occupations, including cinema visits, escape room experiences, walking, air hockey, museum visits, bowling, paintball, shooting, and karting

Session 12: reflection and planning for the future
(i) Reflecting on and discussing the concepts and key points learned during the leisure intervention program (ii) Reflecting on and discussing the barriers and facilitators of participation in leisure activities (iii) Providing solutions to improve participation in leisure activities (iv) Planning for future participation in leisure activities (v) Note: the specific activities listed in sessions 3-11 were chosen as examples and may vary based on individual preferences and availability

**Table 2:** Demographic characteristics of study participants.

Characteristic	Value
Age (years)	30.11 (7.99)
Duration of drug use (years)	4.66 (2)
Abstinence time (months)	21 (10.75)
Sex	
Male (*N*, %)	9 (100%)
Type of drug	
Methamphetamine (*N*, %)	1 (11.1%)
Opiate (*N*, %)	2 (22.2%)
Polysubstance (*N*, %)	6 (66.7%)
Education level	
Subdiploma (*N*, %)	4 (44.4%)
Diploma (*N*, %)	2 (22.2%)
Academic (*N*, %)	3 (33.3%)
Employment status	
Jobless (*N*, %)	4 (44.4%)
Full-time job (*N*, %)	2 (22.2%)
Part-time job (*N*, %)	3 (33.3%)

**Table 3:** Outcome measure scores for preintervention, postintervention, and follow-up assessments.

Measure	Preintervention		Postintervention		Follow-up		Chi-square	*p* value
	Mean (SD)	Median	Mean (SD)	Median	Mean (SD)	Median		
COPM-P	3.44 (0.52)	3.4	6.77 (0.68)	6.8	6.66 (0.70)	6.8	13.556	<0.001
COPM-S	3.32 (0.65)	3.1	6.28 (0.95)	6.2	6.42 (0.60)	6.4	13.557	<0.001
OBQ	7.88 (3.72)	7	15.11 (2.66)	15	14.88 (1.76)	15	14.727	<0.001
NLQ	14 (3.57)	13	25.33 (4.47)	25	24.66 (5.24)	23	13.886	<0.001
SF-36	37.80 (4.76)	46	55.33 (6.03)	53	59.47 (5.48)	58	14.000	<0.001
DDQ	48.77 (5.5)	48	28.11 (4.91)	27	26.66 (6.57)	27	14.000	<0.001

COPM-P: Canadian Occupational Performance Measure-Performance; COPM-S: Canadian Occupational Performance Measure-Satisfaction; OBQ: Occupational Balance Questionnaire; NLQ: Nottingham Leisure Questionnaire; SF-36: 36-Item Short-Form Health Survey; DDQ: Desire to Drug Questionnaire.

**Table 4:** Wilcoxon signed-rank test analysis results.

Measure	Comparison	*z*-score	*p* value	*r*
COPM-P	Preintervention vs. postintervention	-2.677	0.007	0.59
	Preintervention vs. follow-up	-2.675	0.007	0.59
	Postintervention vs. follow-up	-0.789	0.435	0.17

COPM-S	Preintervention vs. postintervention	-2.668	0.008	0.59
	Preintervention vs. follow-up	-2.675	0.007	0.59
	Postintervention vs. follow-up	-0.657	0.511	0.14

OBQ	Preintervention vs. postintervention	-2.673	0.008	0.59
	Preintervention vs. follow-up	-2.692	0.007	0.60
	Postintervention vs. follow-up	-0.333	0.739	0.07

NLQ	Preintervention vs. postintervention	-2.677	0.007	0.59
	Preintervention vs. follow-up	-2.670	0.008	0.59
	Postintervention vs. follow-up	-0.494	0.621	0.11

SF-36	Preintervention vs. postintervention	-2.666	0.008	0.59
	Preintervention vs. follow-up	-2.666	0.008	0.59
	Postintervention vs. follow-up	-1.718	0.086	0.38

DDQ	Preintervention vs. postintervention	-2.675	0.007	0.59
	Preintervention vs. follow-up	-2.666	0.008	0.59
	Postintervention vs. follow-up	-0.773	0.439	0.17

COPM-P: Canadian Occupational Performance Measure-Performance; COPM-S: Canadian Occupational Performance Measure-Satisfaction; OBQ: Occupational Balance Questionnaire; NLQ: Nottingham Leisure Questionnaire; SF-36: 36-Item Short-Form Health Survey; DDQ: Desire to Drug Questionnaire.

## Data Availability

The data used to support this study's findings are available from the corresponding author upon request.

## References

[1] Jones C. M., McCance-Katz E. F. (2019). Co-occurring substance use and mental disorders among adults with opioid use disorder. *Drug and Alcohol Dependence*.

[2] Farhadian M., Akbarfahimi M., Abharian P. H., Hosseini S. G., Shokri S. (2017). Assessment of executive functions in methamphetamine-addicted individuals: emphasis on duration of addiction and abstinence. *Basic and Clinical Neuroscience*.

[3] Cooper R. (2018). *Diagnosing the Diagnostic and Statistical Manual of Mental Disorders*.

[4] Law M. (2002). Participation in the occupations of everyday life. *The American Journal of Occupational Therapy*.

[5] Wasmuth S., Crabtree J. L., Scott P. J. (2014). Exploring addiction-as-occupation. *British Journal of Occupational Therapy*.

[6] Stone M. (2017). Understanding the Impact of Substance Abuse on Occupation Using the Lifestyle History Questionnaire. *Occupational Therapy Doctorate Capstone Projects*.

[7] Stoffel V. C., Moyers P. A. (2004). An evidence-based and occupational perspective of interventions for persons with substance-use disorders. *The American Journal of Occupational Therapy*.

[8] Rawat H., Petzer S. L., Gurayah T. (2021). Effects of substance Use Disorder on Women's roles and Occupational Participation. *South African Journal of Occupational Therapy*.

[9] Roley S., DeLany J. V., Barrows C., Honaker D., Sava D., Talley V. (2008). Occupational therapy practice framework: domain & process 2nd edition. *AJOT: American Journal of Occupational Therapy*.

[10] Jeong E.-H., Park J.-H. (2020). The relationship among leisure activities, depression and quality of life in community-dwelling elderly Koreans. *Gerontology and Geriatric Medicine*.

[11] Ertüzün E., Lapa T. Y. (2020). Relationship between adolescents’ leisure boredom and substance use in Turkey. *Turkish Journal of Sport and Exercise*.

[12] Harmon J. (2018). Leisure participation, substance abuse disorders, and recovery. *Annals of Leisure Research*.

[13] Hawkins J. D., Catalano R. F. (1985). Aftercare in drug abuse treatment. *International Journal of the Addictions*.

[14] Esch T., Stefano G. B. (2004). The neurobiology of pleasure, reward processes, addiction and their health implications. *Neuroendocrinology Letters*.

[15] Gutman S. A., Schindler V. P. (2007). The neurological basis of occupation. *Occupational Therapy International*.

[16] Ikiugu M. N., Lucas-Molitor W., Feldhacker D. (2019). Guidelines for occupational therapy interventions based on meaningful and psychologically rewarding occupations. *Journal of Happiness Studies*.

[17] Law M., Baptiste S., McColl M., Opzoomer A., Polatajko H., Pollock N. (1990). The Canadian occupational performance measure: an outcome measure for occupational therapy. *Canadian Journal of Occupational Therapy*.

[18] Håkansson C., Wagman P., Hagell P. (2020). Construct validity of a revised version of the Occupational Balance Questionnaire. *Scandinavian Journal of Occupational Therapy*.

[19] Drummond A. E., Parker C., Gladman J. R., Logan P., TOTAL Study Group (2001). Development and validation of the Nottingham Leisure Questionnaire (NLQ). *Clinical Rehabilitation*.

[20] Altintas E., Guerrien A., Vivicorsi B., Clément E., Vallerand R. J. (2018). Leisure activities and motivational profiles in adaptation to nursing homes. *Canadian Journal on Aging/La Revue Canadienne du Vieillissement*.

[21] Motamed N., Ayatollahi A., Zare N., Sadeghi H. A. (2005). Validity and reliability of the Persian translation of the SF-36 version 2 questionnaire. *EMHJ-Eastern Mediterranean Health Journal*.

[22] Hassani-Abharian P., Mokri A., Ganjgahi H., Oghabian M.-A., Ekhtiari H. (2016). Validation for Persian versions of “desire for drug questionnaire” and “obsessive compulsive drug use scale” in heroin dependents. *Archives of Iranian Medicine*.

[23] Grissom R. J., Kim J. J. (2012). *Effect Sizes for Research: Univariate and Multivariate Applications*.

[24] Cohen J. (1992). A power primer. *Psychological Bulletin*.

[25] Iwasaki Y., Coyle C., Shank J. (2014). Role of leisure in recovery from mental illness. *American Journal of Psychiatric Rehabilitation*.

[26] Pöllänen S. H. (2015). Crafts as leisure-based coping: craft makers’ descriptions of their stress-reducing activity. *Occupational Therapy in Mental Health*.

[27] Kleiber D. A., Hutchinson S. L., Williams R. (2002). Leisure as a resource in transcending negative life events: self-protection, self-restoration, and personal transformation. *Leisure Sciences*.

[28] Litwiller F., White C., Gallant K. A. (2017). The benefits of recreation for the recovery and social inclusion of individuals with mental illness: an integrative review. *Leisure Sciences*.

[29] Keesmaat S. (1998). How does therapeutic recreation apply in the treatment of addictions?. *Global Therapeutic Recreation*.

[30] Caldwell L. L., Baldwin C. K., Walls T., Smith E. (2004). Preliminary effects of a leisure education program to promote healthy use of free time among middle school adolescents. *Journal of Leisure Research*.

[31] Sivan A., Stebbins R. A. (2011). Leisure education: definition, aims, advocacy, and practices–are we talking about the same thing (s)?. *World Leisure Journal*.

[32] Diener E., Suh E. M., Lucas R. E., Smith H. L. (1999). Subjective well-being: three decades of progress. *Psychological Bulletin*.

[33] Lee H.-Y., Yu C.-P., Wu C.-D., Pan W.-C. (2018). The effect of leisure activity diversity and exercise time on the prevention of depression in the middle-aged and elderly residents of Taiwan. *International Journal of Environmental Research and Public Health*.

[34] Mason P., Curl A., Kearns A. (2016). Domains and levels of physical activity are linked to adult mental health and wellbeing in deprived neighbourhoods: a cross-sectional study. *Mental Health and Physical Activity*.

[35] Sayette M. A., Shiffman S., Tiffany S. T., Niaura R. S., Martin C. S., Schadel W. G. (2000). The measurement of drug craving. *Addiction*.

[36] Helbig K., McKay E. (2003). An exploration of addictive behaviours from an occupational perspective. *Journal of Occupational Science*.

[37] Hodgson S., Lloyd C., Schmid T. (2001). The leisure participation of clients with a dual diagnosis. *British Journal of Occupational Therapy*.

